# Rhein-8-O-β-D-glucopyranoside inhibited high glucose-induced apoptosis of human mesangial cells by regulating the lincRNA ANRIL/let-7a/TGF-β1/Smad signaling pathway

**DOI:** 10.3892/etm.2020.8544

**Published:** 2020-02-24

**Authors:** Lan-Sheng Zhang, Jing Li, Liu Jia-Ping

**Affiliations:** School of Pharmacy and Chemistry, Dali University, Dali, Yunnan 671000, P.R. China

**Keywords:** Rhein-8-O-β-D-glucopyranoside, high glucose, human mesangial cells, long intervening non-coding RNA ANRIL, transforming growth factor-β1/Smad signaling pathway

## Abstract

Diabetic nephropathy is one of most frequent complications of diabetes, and is the major cause of end-stage disease in diabetic patients. The present study investigated the roles and mechanisms of Rhein-8-O-β-D-glucopyranoside (Rg) protecting human mesangial cells (HMCs) from high glucose (HG)-induced apoptosis. Using a Cell Counting Kit-8 assay the proliferation of HMCs was analyzed, and flow cytometry was applied to detect apoptosis. The apoptosis-associated protein Bcl-2, caspase-3 and members of the transforming growth factor-β1 (TGF-β1)/Smad signaling pathway were analyzed using a western blotting assay. HG significantly induced HMC apoptosis, and Rg markedly attenuated the HG-induced apoptosis. HG decreased the Bcl-2 expression and increased the caspase-3 expression, and Rg treatment recovered the expressions of Bcl-2 and caspase-3 affected by HG. The underlying mechanisms were further analyzed, and it was demonstrated that HG significantly upregulated the long intervening non-coding RNA (lincRNA) ANRIL expression level, downregulated let-7a expression and activated the TGF-β1/Smad signaling pathway; Rg treatment recovered the expressions of lincRNA ANRIL and let-7a, and inhibited the TGF-β1/Smad signaling pathway in the condition of HG. In conclusion, the present results suggested that Rg attenuated HG-induced apoptosis of HMCs by regulating the lincRNA ANRIL/let-7a/TGF-β1/Smad signaling pathway.

## Introduction

Diabetic nephropathy (DN) is one of most frequent complications of diabetes, and is the major cause of end-stage disease in diabetic patients (1). DN is characterized by mesangial expansion, glomerular extracellular matrix (ECM) accumulation and renal interstitial fibrosis, and these pathological changes lead to chronic renal dysfunction (2). Human mesangial cells (HMCs) which produce mesangial ECM constituents are located in the interpapillary space of the glomerular tufts (3).

Ye *et al* (4) reported that Norcantharidin could inhibit HMC proliferation and induce apoptosis in a dose and time-dependent manner. Zhou *et al* (5) observed that mevalonate could stimulate HMC proliferation, increase the expression of Bcl-2 and downregulate the expression of Bax in the HMCs. The present study demonstrated that Rhein-8-O-β-D-glucopyranoside (Rg) could significantly inhibit high glucose (HG)-induced HMC apoptosis; however, the underlying mechanisms were largely unknown.

A previous study demonstrated that transforming growth factor-β (TGF-β) plays a key role in the progression of DN (6). Smad2, a member of receptor Smads, is phosphorylated when TGF-β1 binds to TGF-β receptor. Smad7, which is an inhibitory Smad, could bind to type I receptors and prevent phosphorylation of receptor Smads (7). The TGF-β1/Smad signaling pathway was activated in DN, and could also be induced by HG treatment in HMCs (8). The present study further investigated the roles of the TGF-β1/Smad signaling pathway in Rg-treated HMCs.

In the present study, the roles and mechanisms of Rg on HG-induced apoptosis of HMCs were examined. The present study suggested that Rg alleviated HG-induced apoptosis of HMCs, and Rg increased HG-reduced Bcl-2 expression and decreased HG-induced caspase-3 expression. Rg inhibited the HG-activated TGF-β1/Smad signaling pathway by regulating long intervening non-coding RNA (lincRNA) ANRIL/let-7a expressions.

## Materials and methods

### 

#### Purification of Rg

Rhubarb was purchased from Tong Ren Tang Technologies Co., Ltd (http://www.tongrentangkj.com). Rg was extracted from rhubarb according to the method from a previous study (9).

#### Cell culture and transfection

HMCs were purchased from ScienCell Research Laboratories, Inc. and cultured in Dulbecco's modified Eagle's medium (DMEM; Gibco; Thermo Fisher Scientific, Inc.) with 10% FBS (Gibco; Thermo Fisher Scientific, Inc.), penicillin (100 U/ml) and streptomycin (100 mg/ml). High glucose culture media was made by supplementing normal DMEM medium with additional D-glucose at a final concentration of 25 mM (HG). All of these cells were maintained at 37˚C with 5% CO_2_.

Cells were seeded in the six-well plates at a density of 5x10^5^ cells per well and treated with Rg (20 or 80 µM) or transfected with lincRNA ANRIL small interfering (siRNA; 50 nM), let-7a mimics (50 nM) or negative control siRNA (50 nM) using Lipofectamine^®^ 3000 transfection reagent (Invitrogen; Thermo Fisher Scientific, Inc.) and cultured using HG DMEM medium according to the manufacturer's protocol at 37˚C for 48 h. The lincRNA ANRIL siRNA sequence was 5'-GGUCAUCUCAUUGCUCUAU-3', and let-7a mimics sequence was 5'-UGAGGUAGUAGGUUGUAUAGUU-3', and negative control siRNA sequence was 5'-UUCUCCGAACGUGUCACGUTT-3'. The lincRNA ANRIL siRNA, let-7a mimics and negative control were synthesized by Shanghai GenePharma Co., Ltd.

#### CCK-8 assay

Cell proliferation was detected using a Cell Counting Kit-8 (CCK-8; Dojindo Molecular Technologies, Inc.) as previously described (10).

#### Flow cytometry assay

Apoptosis was detected using flow cytometry. In apoptosis assay, harvested cells were double-stained with Annexin V (room temperature for 15 min) and propidium iodide (PI; room temperature for 5 min) according to the protocol of a FITC-Annexin V cell apoptosis assay kit (BD Biosciences). Then the cells were analyzed using a flow cytometer (FACScan; BD Biosciences) equipped with CellQuest pro software (v5.2, BD Biosciences).

#### RNA extraction and reverse transcription-quantitative PCR (RT-qPCR) assay

RNA of cells was extracted using a Total RNA Rapid Extraction kit (BioTeke Corporation) according to the manufacturer's protocol. After detecting the concentration, 1 µg RNA sample was reverse transcribed into cDNA with M-MLV reverse transcriptase (BioTeke Corporation) in the presence of oligo(dT) and 50 random primers (Invitrogen; Thermo Fisher Scientific, Inc.). The RT procedure is 42˚C for 60 min, and 70˚C for 10 min. The instruments used for this experiment were pre-treated using surface RNase Erase (Tiandz, Inc.) and the reagents were RNase-free. The cDNA (1 µl each reaction) was used for real-time PCR to detect the gene expressions using 2X Power Taq PCR MasterMix (BioTeke Corporation) and SYBR Green (Beijing Solarbio Science & Technology Co., Ltd.), with GAPDH as the internal control. The PCR procedure was as follows: 95˚C for 10 min, 38 cycles of 95˚C for 12 sec, 60˚C for 18 sec and 72˚C for 30 sec, and finally 4˚C for 5 min. Calculations were performed using the 2^-^^ΔΔ^^Cq^ method (11). The following primer pairs were used for amplification: ANRIL forward, 5'-GGACTACAGATGCACCACCAT-3', ANRIL reverse, 5'-TGAGCACTGTGTCCATAGCA-3'; GAPDH forward, 5'-AAATCCCATCACCATCTTCCAG-3', and GAPDH reverse, 5'-GAGTCCTTCCACGATACCAAAGTTG-3'.

Hairpin-it^™^ let-7a RT-qPCR Primer Set (Shanghai GenePharma Co., Ltd.) was used for the measurement of the relative quantity of let-7a. The reaction conditions were as follows: 95˚C for 4 min, 30 cycles of 95˚C for 30 sec, 57˚C for 30 sec and 72˚C for 30 sec. The mRNA expression of let-7a was normalized to the endogenous expression of U6. The primer sequences were as follows: Let-7a forward: 5'-CACCCACCACTGGGAGATAAC-3', and let-7a reverse, 5'-TATGGTTGTTCACGACTCCTTCAC-3'; U6 forward: 5'-GCTTCGGCAGCACATATACTAAAAT-3', and U6 reverse, 5'-CGCTTC.ACGAATTTGCGTGTCAT-3'.

#### Western blot analysis

Protein was extracted using a whole-cell lysis kit (CWBio) from cells and the concentration of protein was measured using a BCA protein quantitative kit (Beyotime Institute of Biotechnology). After being denatured by boiling, the protein sample (40 µg for each lane) was separated by 10% SDS-PAGE and transferred to a PVDF membrane (EMD Millipore). After blocking with 5% skim milk (Inner Mongolia Yili Industrial Group Co., Ltd.) at room temperature for 1 h, the membrane was incubated with the primary antibodies at 4˚C overnight. After rinsing with TBS with Tween-20, the membrane was incubated with goat anti-rabbit Ig G labeled with horseradish peroxidase (HRP; cat. no. sc-2004; 1:5,000; Santa Cruz Biotechnologies, Inc.) or goat anti-mouse Ig G-HRP (cat. no. sc-2005; 1:5,000; Santa Cruz Biotechnologies, Inc.) at 37˚C for 45 min, and exposed with ECL reagent (Thermo Fisher Scientific, Inc.). Optical density values of bands were analyzed using a gel image processing system ImageLab software (version: 3.0, Bio Rad Laboratories, Inc.). The primary antibodies were as follows: Bcl-2 antibody (cat. no. ab185002; 1:1,000; Abcam); cleaved caspase-3 antibody (cat. no. ab2302; 1:1,000; Cell Signaling Technology, Inc.); TGF-β1 antibody (cat. no. ab92486; 1:1,000; Abcam); phosphorylated (p)-Smad2 antibody (cat. no. ab188334; 1:1,000; Abcam); Smad2 antibody (cat. no. ab33875; 1:1,000; Abcam); Smad7 antibody (cat. no. ab216428; 1:1,000; Abcam); and β-actin antibody (cat. no. ab179467; 1:1,000; Abcam).

#### Statistical analysis

Statistical analyses were performed using GraphPad Prism 6 (GraphPad Software, Inc.). The data in the present study are presented as the mean ± SD of three or five individual experiments, and analyzed by one-way ANOVA followed by Tukey's multiple comparison test. P<0.05 was considered to indicate a statistically significant difference.

## Results

### 

#### Rg inhibits HG-induced apoptosis and promotes HG-suppressed growth of HMCs

**Figure 1:**
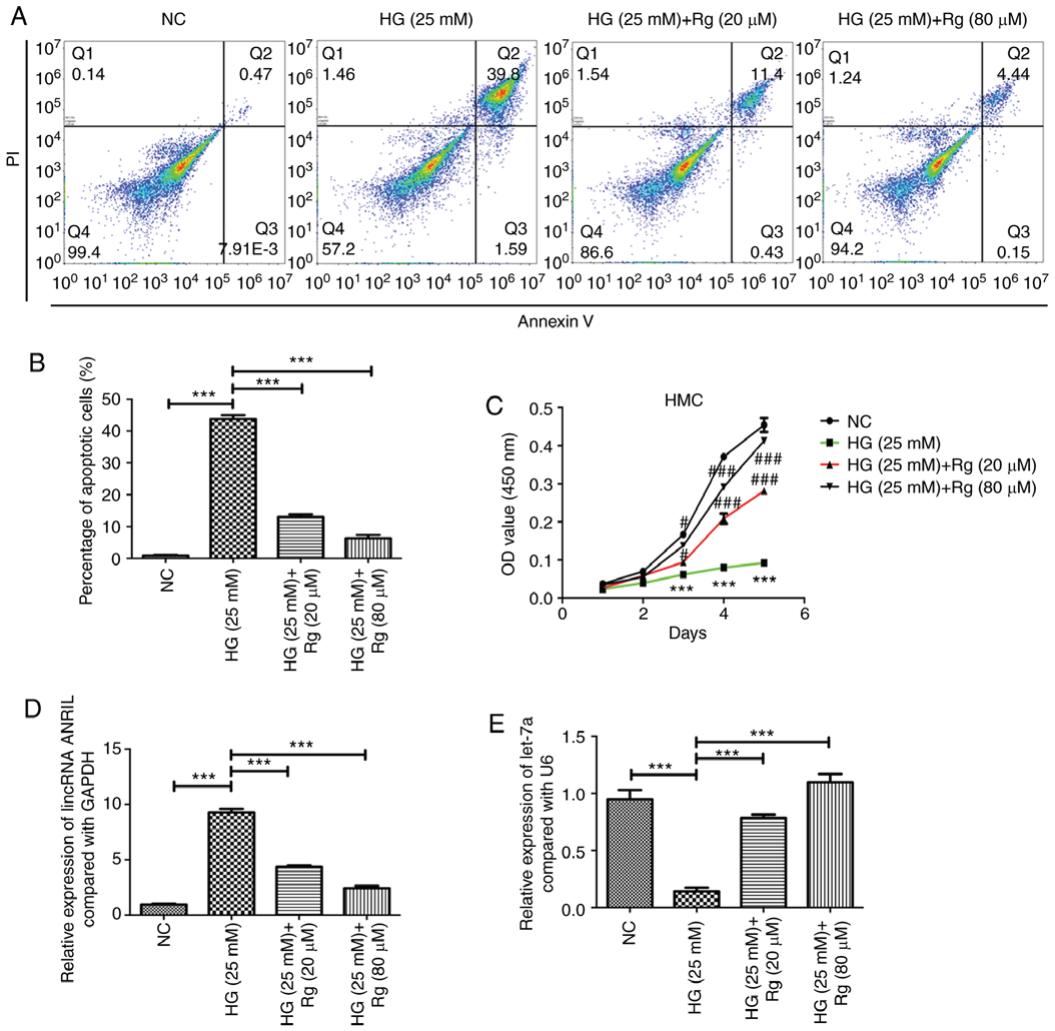
Rg inhibits HG-induced apoptosis and promotes HG-suppressed growth of HMCs. (A) Apoptosis of HMCs was detected using a flow cytometry method. (B) Statistical analysis of flow cytometry results. ^*^^*^^*^P<0.001. (C) Proliferation of cells was detected using a Cell Counting Kit-8 assay. ^*^^*^^*^P<0.001 vs. NC; ^#^P<0.05, ^###^P<0.001 vs. HG. Reverse transcription-quantitative PCR analysis was applied to detect the expressions of (D) lincRNA ANRIL and (E) let-7a. ^*^^*^^*^P<0.001. HG, high glucose; RG, Rhein-8-O-β-D-glucopyranoside; lincRNA, long intervening non-coding RNA; OD, optical density; NC, negative control; PI, propidium iodide; HMC, human mesangial cell.

HMCs were cultured in HG (25 mM) DMEM for 24 h, and then treated with Rg for 48 h. The results showed that HG significantly induced the apoptosis of HMCs, and 20 and 80 µM Rg could both inhibit the apoptosis induced by HG (Fig. 1A and B). Using a Cell Counting Kit-8 assay, it was also observed that HG significantly suppressed the cell proliferation of HMCs, and 20 and 80 µM Rg could both promote the cell growth, which was inhibited by HG (Fig. 1C).

#### Rg reduces HG-induced lincRNA ANRIL expression, increases HG-reduced let-7a expression and inhibits the HG-activated TGF-β/Smad signaling pathway

A previous study reported that HG and diabetes could upregulate lincRNA ANRIL in human retinal endothelial cells and in the retina, and lincRNA ANRIL could also regulate vascular endothelial growth factor expression (12). The present study examined whether HG regulates the expression of lincRNA ANRIL in HMCs, and whether Rg inhibits the apoptosis of HMCs induced by HG through lincRNA ANRIL. The present study demonstrated that HG significantly upregulated the expression of lincRNA ANRIL, and 20 and 80 µM Rg decreased the HG-induced lincRNA ANRIL expression (Fig. 1D). HG decreased the expression of let-7a, and 20 and 80 µM Rg could both increase the HG-reduced let-7a expression (Fig. 1E).

**Figure 2:**
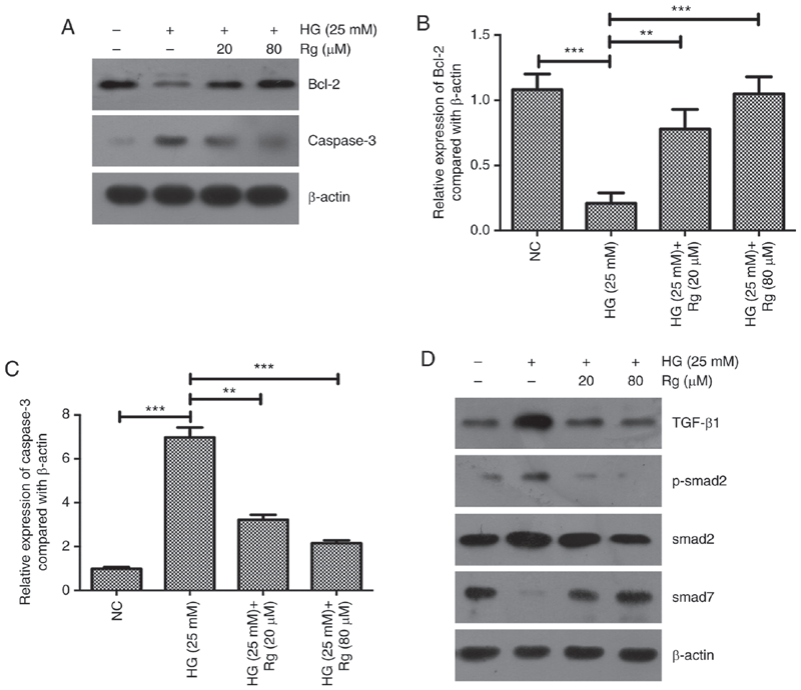
Rg inhibits HG-activated TGF-β/Smad signaling. (A) Western blotting and subsequent densitometry were performed to analyze the protein expressions of (B) Bcl-2 and (C) caspase-3. (D) Western blotting and subsequent densitometry were performed to detect the protein expressions of (E) TGF-β1, (F) p-Smad2/Smad2 and (G) Smad7. ^*^^*^P<0.01, ^*^^*^^*^P<0.001. HG, high glucose; RG, Rhein-8-O-β-D-glucopyranoside; TGF-β, transforming growth factor-β; NC, negative control; p, phosphorylated.

The expressions of apoptosis-associated proteins Bcl-2 and active caspase-3 were further detected. The present results showed that HG significantly inhibited Bcl-2 expression and increased active caspase-3 expression, and Rg treatment recovered the expressions of Bcl-2 and caspase-3 affected by HG (Fig. 2A-C). The present results suggested that Rg inhibited the apoptosis of HMCs induced by HG by upregulating Bcl-2 and downregulating caspase-3.

Previous studies demonstrated that dencichine could ameliorate kidney injury in induced type II DN via the TGF-β/Smad signaling pathway and 1,25(OH)2D3/VDR could attenuate HG-induced epithelial-mesenchymal transition in human peritoneal mesothelial cells via the TGF-β/Smad3 pathway (13,14). The present study aimed to determine whether the TGF-β/Smad signaling pathway is involved in the regulation of Rg on the HG-affected cell apoptosis and proliferation. Using a western blotting assay, HG significantly upregulated TGF-β1 expression, downregulated Smad7 expression and activated the phosphorylation of Smad2. Both 20 and 80 µM Rg treatment recovered the expression levels of TGF-β1 and Smad7, and the phosphorylation status of Smad2 regulated by HG (Fig. 2D-G).

#### Knockdown of lincRNA ANRIL and overexpression of let-7a have similar effects to Rg on HG-induced apoptosis and HG-suppressed growth of HMCs

**Figure 3:**
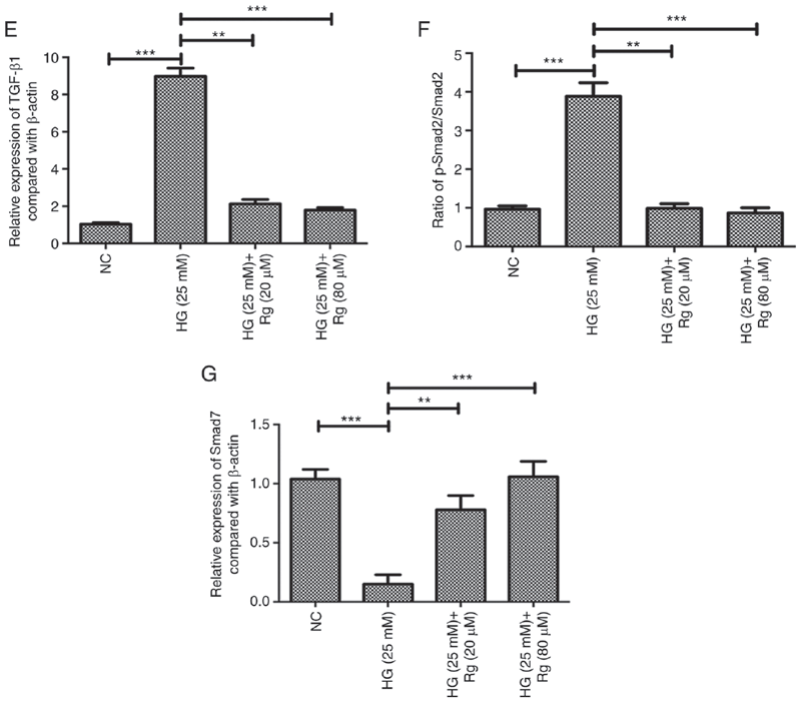
Knockdown of lincRNA ANRIL and overexpression of let-7a have similar effects to Rg on HG-induced apoptosis and HG-suppressed growth of human mesangial cells. (A) Efficacy of lincRNA ANRIL knockdown and let-7a overexpression was confirmed by RT-qPCR. (B) Apoptosis of human mesangial cells in different treatments was detected using a flow cytometry method. (C) Statistical analysis of flow cytometry results. RT-qPCR analysis was applied to detect the expressions of (D) lincRNA ANRIL and (E) let-7a. ^*^^*^^*^P<0.001. lincRNA, long intervening non-coding RNA; HG, high glucose; RG, Rhein-8-O-β-D-glucopyranoside; NC, negative control; RT-qPCR, reverse transcription-quantitative PCR; PI, propidium iodide; siRNA, small interfering RNA.

To further confirm whether lincRNA ANRIL and let-7a are involved in regulation of cell apoptosis and the TGF-β1/Smad signaling pathway by Rg treatment in the condition of HG, the present study detected whether knockdown of lincRNA ANRIL and overexpression of let-7a had the same results as Rg treatment. RT-qPCR was used to confirm the efficacy of lincRNA ANRIL knockdown and let-7a overexpression (Fig. 3A). The results showed that knockdown of lincRNA ANRIL and overexpression of let-7a significantly inhibited HG-induced apoptosis, similar to Rg treatment (Fig. 3B and C). Overexpression of let-7a had no effect on the expression of lincRNA ANRIL in an HG condition (Fig. 3D), but knockdown of lincRNA ANRIL significantly upregulated the level of let-7a compared with the HG group (Fig. 3E). The present results suggested that lincRNA ANRIL could negatively regulate let-7a in an HG condition and that lincRNA ANRIL was an upstream regulator of let-7a.

#### Knockdown of lincRNA ANRIL and overexpression of let-7a have similar effects to Rg on apoptosis-associated proteins and the TGF-β1/Smad signaling pathway in an HG condition

**Figure 4:**
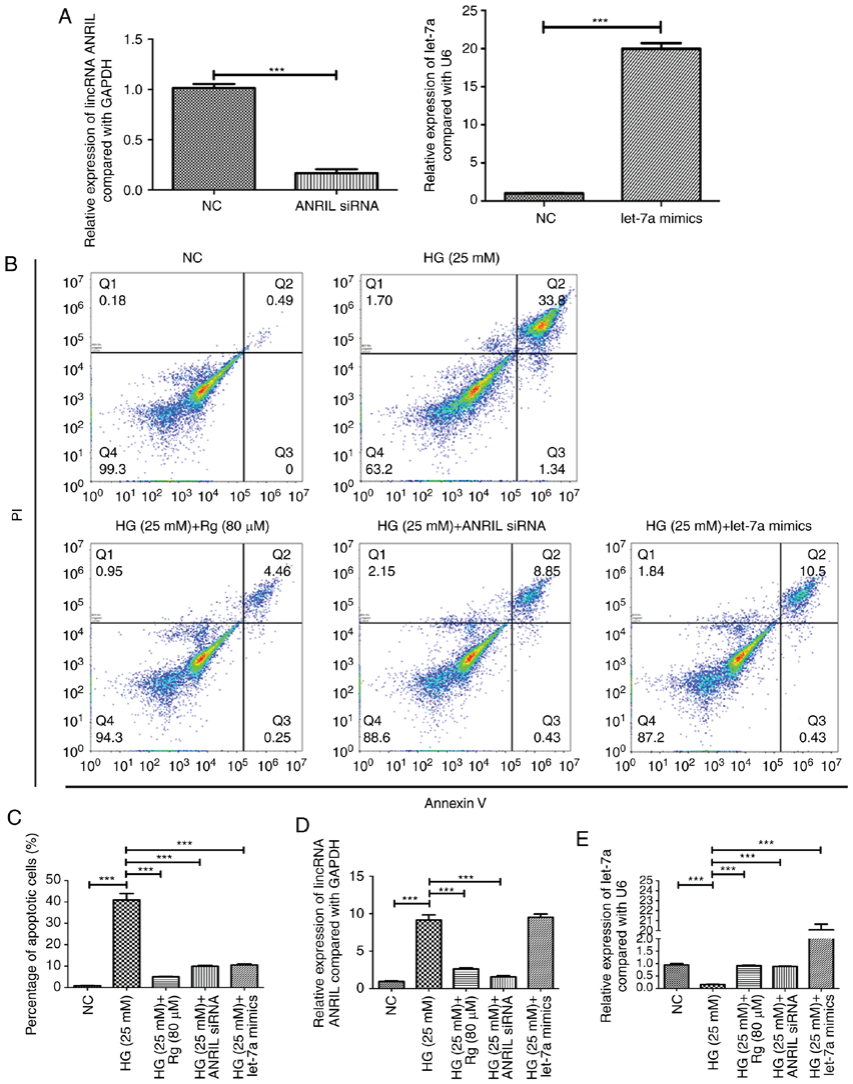
Knockdown of long intervening non-coding RNA ANRIL and overexpression of let-7a have similar effects to Rg on apoptosis-associated proteins and the TGF-β1/Smad signaling pathway in an HG condition. (A) Western blotting and subsequent densitometry were performed to analyze the protein expressions of (B) Bcl-2 and (C) caspase-3. (D) Western blotting and subsequent densitometry were performed to detect the protein expressions of (E) TGF-β1, (F) p-Smad2/Smad2 and (G) Smad7. ^*^^*^^*^P<0.001. HG, high glucose; RG, Rhein-8-O-β-D-glucopyranoside; TGF-β, transforming growth factor-β; NC, negative control; p, phosphorylated; siRNA, small interfering RNA.

Knockdown of lincRNA ANRIL and overexpression of let-7a significantly increased the HG-suppressed Bcl-2 expression and decreased the HG-induced caspase-3 expression, similar to the Rg treatment (Fig. 4A-C). Knockdown of lincRNA ANRIL and overexpression of let-7a significantly reduced the HG-induced TGF-β1 level, inhibited the HG-activated phosphorylation of Smad2, and increased the HG-reduced Smad7 level (Fig. 4D-G). The present results suggested that Rg attenuated HG-induced apoptosis by regulating the lincRNA ANRIL/let-7a/TGF-β1/Smad signaling pathway.

## Discussion

The present study hypothesized that Rg inhibited HG-induced apoptosis of HMCs by inactivating the TGF-β1/Smad signaling pathway. The present results showed that HG significantly induced HMC apoptosis and Rg markedly attenuated the HG-induced apoptosis. HG was also demonstrated to decrease the Bcl-2 expression and increase the caspase-3 expression, and Rg treatment recovered the expressions of Bcl-2 and caspase-3 affected by HG. The underlying mechanisms were further examined and it was observed that HG significantly upregulated the lincRNA ANRIL level, downregulated let-7a expression and activated the TGF-β1/Smad signaling pathway; Rg treatment recovered the expressions of lincRNA ANRIL and let-7a, and inhibited the TGF-β1/Smad signaling pathway in an HG condition.

The TGF-β1/Smad signaling pathway plays a key role in the progression of DN. Xie *et al* (15) observed that relaxin inhibited HG-induced matrix accumulation in HMCs by interfering with TGF-β1 production and mesangial cells phenotypic transition; telmisartan activated peroxisome proliferator-activated receptor-σ and had anti-fibrotic effects in HMCs. The present results suggested that Rg treatment could inhibit the HG-activated TGF-β1/Smad signaling pathway in HMCs.

The present study further investigated the mechanisms underlying the regulation between Rg treatment and the TGF-β1/Smad signaling pathway. The lincRNA ANRIL, which is associated with atherosclerosis, periodontitis and several types of cancer, was reported to regulate adiponectin 1, vesicle associated membrane protein 3 and chromosome 11 open reading frame 10(16). In nasopharyngeal carcinoma, lincRNA ANRIL was upregulated, and could promote cancer progression by increasing proliferation, reprograming cell glucose metabolism and inducing side-population stem-like cancer cells (17). However, the role and mechanism of lincRNA ANRIL in HG-induced HMC apoptosis and Rg treatment are still unknown to the best of the authors' knowledge. let-7a was downregulated in both rats with DN and mesangial cells in an HG condition, and could negatively regulate the expression of TGFβ receptor 1(18). Katayama *et al* (19) reported that glucose could significantly regulate the expression of let-7a by affecting promoter activity. Wang *et al* (20) found that lincRNA ANRIL could negatively regulate the expression of let-7a. The present study demonstrated that HG could upregulate lincRNA ANRIL expression and downregulate the level of let-7a, and Rg treatment decreased HG-induced lincRNA ANRIL expression and increased HG-suppressed let-7a expression. Knockdown of lincRNA ANRIL upregulated let-7a expression. The present results suggested that Rg treatment recovered lincRNA ANRIL and let-7a expression in an HG condition. Although the current study indicated that Rg attenuated HG-induced HMC apoptosis by regulating the ANRIL/let-7a axis and the downstream TGF-β1/Smad signaling pathway, the mechanism of how ANRIL/let-7a axis regulated TGF-β1/Smad signaling pathway is still yet to be determined.

In conclusion, Rg inhibited HG-induced apoptosis by regulating the lincRNA ANRIL/let-7a/TGF-β1/Smad signaling pathway. In the future, Rg may be developed as a new therapeutic method in diabetic nephropathy.

## Data Availability

All data generated or analyzed during this study are included in this published article.
